# Integration of metabolomics and transcriptomics to reveal anti-immunosuppression mechanism of *Lycium barbarum* polysaccharide

**DOI:** 10.3389/fphar.2024.1486739

**Published:** 2024-11-13

**Authors:** Jiandong Wang, Xue Zhang, Yi Wu, Qianfei Wei, Lingshan Yan, Youli Yu, Yanan Guo, Zhengqin Yu, Pan Wang, Xiaonan Yang

**Affiliations:** ^1^ Institute of Animal Science, Ningxia Academy of Agricultural and Forestry Sciences, Yinchuan, Ningxia, China; ^2^ College of Animal Science and Technology of Yunnan Agricultural University, Kunming, Yunnan, China; ^3^ College of Veterinary Medicine of Yunnan Agricultural University, Kunming, Yunnan, China; ^4^ College of Veterinary Medicine, Nanjing Agricultural University, Nanjing, Jiangsu Province, China; ^5^ College of Agriculture, Ningxia University, Yinchuan, Ningxia, China; ^6^ National Engineering Research Center for Southwest Endangered Medicinal Resources Development, Guangxi Botanical Garden of Medicinal Plants, Nanning, Guangxi, China

**Keywords:** immunosuppression, *Lycium barbarum* polysaccharide, transcriptomics, metabolomics, Mechanism

## Abstract

It is well documented that immunosuppression in chickens increases the risk of secondary infections and immunodeficiencies, resulting in significant financial setbacks for the poultry sector. It is crucial to determine if *Lycium barbarum* polysaccharide (LBP) can counteract immune suppression in young chickens, considering its known ability to modulate immune responses. The aim of this study was to investigate the antagonistic effect and mechanism of LBP on immunosuppression in chicks. A total of 200 seven-day-old Hyland Brown laying hens were used to develop an immunosuppression model and to investigate the optimal time of use and optimal dosage of LBP. A further 120 seven-day-old Hyland Brown laying hens were used to investigate the mechanism of antagonism of LBP against immunosuppression at the optimal time and dosage. The results demonstrated that LBP significantly elevated body weight, spleen index, and peripheral lymphocyte transformation rate, and ameliorated pathological spleen damage in immunosuppressed chickens. A total of 178 differential genes were significantly upregulated following LBP intervention, with a significant enrichment in immune-related pathways, including the chemokine signalling pathway, the C-type lectin receptor signalling pathway, the B-cell receptor signalling pathway, platelet activation, natural killer cell-mediated cytotoxicity, and Th1 and Th2 cell differentiation. A total of 20 different metabolites were identified by metabolomics, which were mainly involved in vitamin metabolism, lipid metabolism, nucleic acid metabolism and amino acid metabolism. The integrated examination of transcriptomic and metabolomic data revealed that the glycerophospholipid metabolic pathway stands out as the most significant among all metabolic pathways. The results demonstrated that LBP regulate the immune system in a multi-pathway and multi-target way.

## 1 Introduction

The causes of impaired immune system function are diverse, including viruses, stress, mycotoxins, and pharmacological factors. Viral diseases such as Marek’s disease virus (MDV), chicken infectious anaemia virus (CIA xV), and avian leukaemia virus (ALV) are common causes of immunosuppression in poultry (Wang et al., 2022). Stress factors such as heat stress and feed mycotoxin contamination also contribute to immunosuppression by reducing the relative weight of the bursa and spleen, reducing the formation of the germinal centre, and thereby reducing the body’s immune regulatory function (Calefi et al., 2016). The current research indicates that mycotoxins can invade the body’s immune system, resulting in damage to immune organs and a reduction in the physiological function of immune cells. This, in turn, leads to a reduction in the production of immune factors such as interleukins and lymphokines, which causes the body to undergo immunosuppression (You et al., 2023). Furthermore, the irrational addition of antibiotics in feed and long-term abuse in daily life will have a certain inhibitory effect on the proliferation and differentiation of immune cells (Jabs, 2018). The aforementioned immunosuppressive factors not only result in mortality but also increase susceptibility to other microbial infections and subsequent failure of vaccination against other diseases, leading to significant economic losses (Ma et al., 2023). There is an urgent need to explore safe and effective drugs to counteract immunosuppression, Chinese herbal medicines have become particularly ideal materials (Abd et al., 2020).

*Lycium barbarum* (LB) is widespread in arid and semi-arid regions of the world and is a member of the Solanaceae family. In China, it is mainly found in northwestern and central China (Cao et al., 2021; Zhang et al., 2023). Recent studies have demonstrated that LBP exhibits both medicinal and food homology (Yu et al., 2023). Several studies have shown that LBP has antioxidant, antitumour, anti-inflammatory, immunopotentiating, reproductive and neuroprotective effects (Ma et al., 2023). LBP is one of its main active components, and previous studies have demonstrated that LBP can affect immune regulation through the modulation of immune cells, such as DC, MA, NK, T, and B lymphocytes (Tian et al., 2019). LBP could promote DCs maturation by mediating Notch signaling (Wang et al., 2018), enhance the functionalof CTL, significantly enhance NO, macrophage capacity *in vivo*, and could enhance production of TNF-A (Gong et al., 2018), indicating the potentially safe anti-immunosuppressive effect of LBP.

Consequently, this study investigated the immunoregulatory effect of LBP on growth status, spleen index, peripheral blood lymphocyte proliferation rate and spleen histology, and then studied the effect of LBP on gene expression and metabolites of RNA-seq and UHPLC-Triple-TOF-MS, analyzing the protective mechanism of LBP in order to provide new insights into the mechanism of LBP antagonism of immunosuppression in chicks.

## 2 Materials and methods

### 2.1 Animal ethics and experimental design

#### 2.1.1 Animal ethics

This study was conducted in accordance with the ethical standards approved by the Ningxia Academy of Agricultural and Forestry Sciences Institutional Animal Care and Use Committee, with the ethical review number NXNKYKJLL-2024-3.

#### 2.1.2 Experimental design

In our preliminary studies, the immune-enhancing effect of LBP after 25 days of administration was found to be superior to that of existing immunomodulators such as astragalus polysaccharides and propolis polysaccharides (Wang et al., 2020). Therefore, in this experiment, a comparison with existing immunomodulators was not designed. The experiment was divided into two parts. The first part was designed to investigate whether LBP had any effect on immunosuppression in Hyland’s brown laying hens. The mechanism of action of LBP in antagonising immunosuppression was elucidated in the part 2.

**FIGURE 1:**
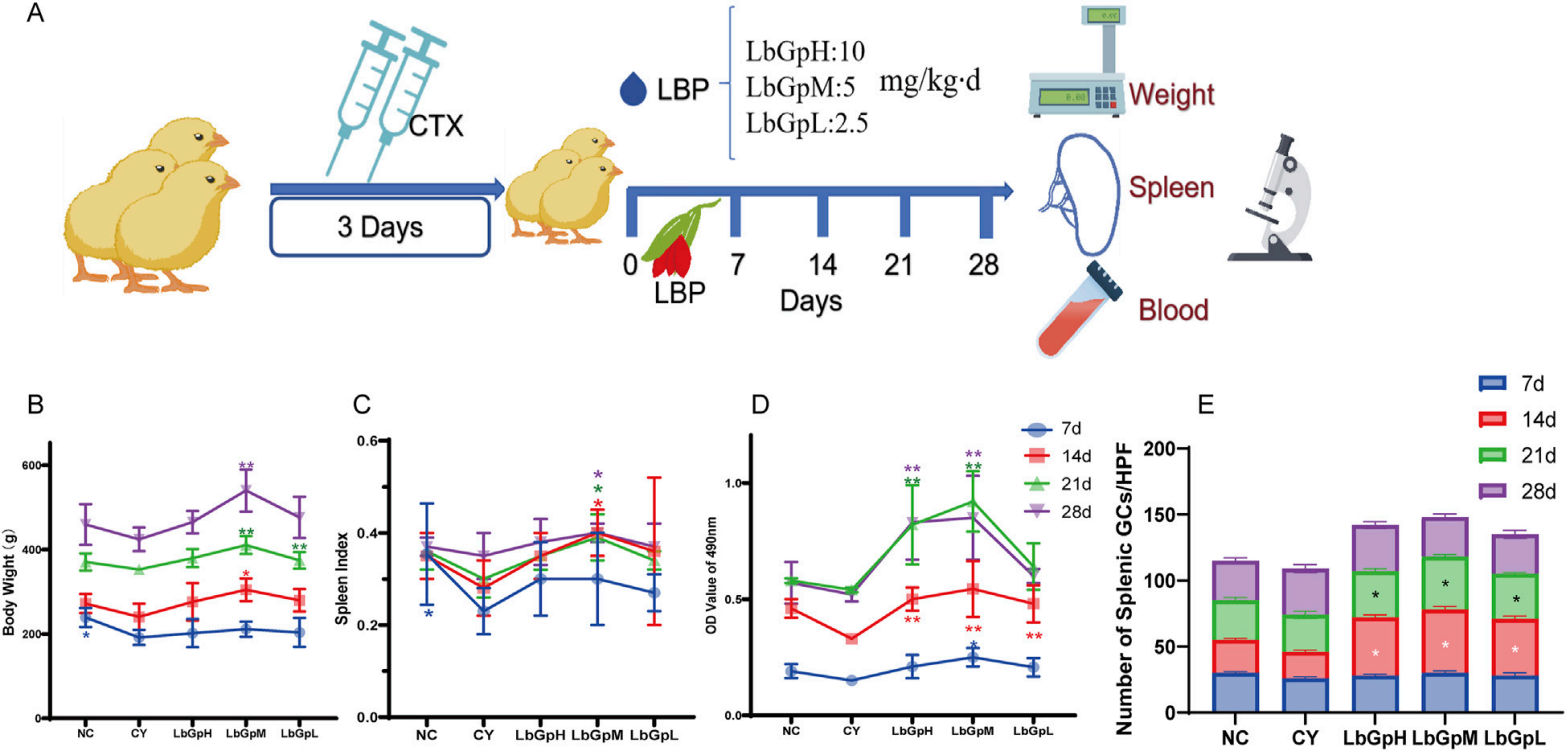
Effect of LBP on cyclophosphamide immunosuppressed chicks. **(A)** Experimental design of Patr1. **(B)** Body weight. **(C)** spleen index. **(D)** Lymphocyte metastasis. **(E)** germinal centers in the spleen.

Part 1: A total of 200 one-day-old Hyland’s Brown laying hens from Huadu Yukou Poultry Co., Ltd., China, were acclimatized to 7 days of age and then randomly divided into five groups (n = 40 per group): NC, CY, LbGpH, LbGpM, and LbGpL. The CY, LbGpH, LbGpM, and LbGpL groups were injected with CTX (cyclophosphamide, 80 mg/kg per day for 3 days, intramuscularly) to establish an immunosuppression model, while the NC group received an equivalent volume of saline for three consecutive days. Subsequently, the LbGpH, LbGpM, and LbGpL groups were given LBP at doses of 10, 5, and 2.5 mg/kg per day via drinking water for 7 consecutive days, post-modeling. Finally, five chickens from each group were selected on the 7th, 14th, 21st, and 28th days after the first LBP administration for measurements of body weight, spleen index, lymphocyte proliferation, and assessment of spleen pathological injury to evaluate the effects of LbGpH, LbGpM, and LbGpL. Experimental design for this part has shown in Figure 1A.

**FIGURE 2:**
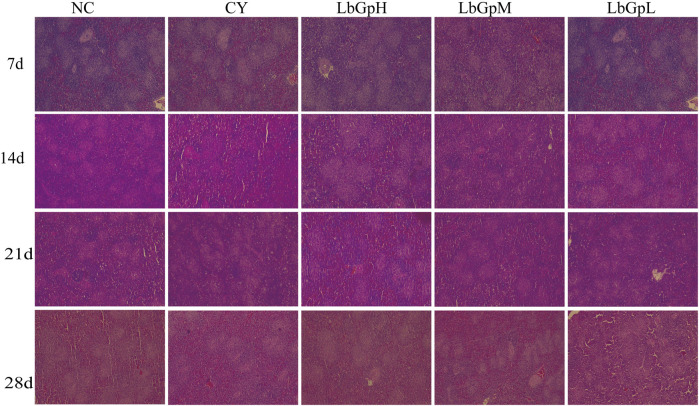
Splenic microscopy was performed.

Part 2: A total of 120 1-day-old Hyland Brown laying hens (Huadu Yukou Poultry Co., Ltd., China) were acclimatised to 7 days of age and then randomly divided into three groups, namely, NC, CY, and CYLbGp, with 40 hens in each group. The NC group was administered saline via intramuscular injection for a period of three consecutive days. The remaining two groups were injected with 80 mg/kg of cyclophosphamide intramuscularly for the same duration. Following the modelling phase, the CYLbGp group was administered 5 mg/kg LBP via drinking water for a period of 30 days. On the first day of LBP administration, six chickens were selected from each group and their spleens were stored at −80°C. The spleens were subsequently used for transcriptome sequencing and metabolic profiling. The spleens were used for transcriptome sequencing and metabolome sequencing. Experimental design for this part has shown in Figure 2A.

### 2.2 Spleen index

In Part1, the spleen was weighed, and the data was subsequently collated for the purpose of calculating the spleen index. The formula is: spleen index = spleen weight (g)/body weight (g) × 100.

### 2.3 Determination of lymphocyte proliferation

In Part 1, the blood has been collected and then isolated lymphocytes. MTT method was used to determine the OD490 mm value of lymphocytes.

### 2.4 Splenic tissue sections

In Part 1, the spleen collected was fixed with 10%NBF, stored at room temperature for 3 days, paraffin sections were prepared according to routine operations, HE staining was performed, and microscopic examination was carried out.

### 2.5 RNA sequencing

Total RNA was extracted from the spleen in part 2 via the Trizol method. The mRNA libraries were prepared in accordance with the instructions provided by the kit manufacturer (Novozymes Biotechnology Co., Ltd., China) and sequenced on the Illumina NovaSeq 6,000 platform, with a read length of 2 × 150 bp. The clean data obtained from the sequencing were subsequently used for the sequencing of the sample with an average base error rate of less than 0.1%. The average error rate of the sequenced clean data bases was less than 0.1%, which was used for subsequent transcriptome analysis. Following the acquisition of the gene read counts, RSEM was employed to transform them into transcripts per million (TPM), thereby facilitating the generation of normalised gene expression levels. The software DESeq2 was employed to identify the genes exhibiting differential expression among the samples within each group |log2 (Fold change| > 0, P-adjust < 0.05). GO annotation analysis was performed on the gene sets using Blast 2 go 2.5, while GO enrichment analysis was conducted on the genes within the gene sets using Goatools. The Kyoto Encyclopedia of Genes and Genomes (KEGG) analysis was performed on the genes within the gene sets via the KOBAS software, with the goal of pinpointing pathways that were markedly enriched with differentially expressed genes.

### 2.6 RT-qPCR validation

RT-qPCR was employed for transcriptome validation, five differentially expressed genes, CD83, CCL4, PSPH, LPIN2, and ULK2, were randomly selected from genes with criteria: gene length >700, read counts >100, FPKM ranged 0.03–100. The RT-qPCR method was employed to verify the accuracy of the transcriptome sequencing results. β-actin was used as the internal reference gene. The primers were synthesised by Meiji Biomedical Technology Co., Ltd. and are listed in Table 1. The RT-qPCR system consisted of 20 μL of 2× ChamQ SYBR Color qPCR Master Mix, 10 μL of each forward and reverse primer, 0.4 μL of cDNA, and ddH_2_O. The reaction conditions were as follows: 95°C for 5 s, 55°C for 30 s, extension at 72°C for 40 s, 40 cycles. Determined the relative expression of the experimental groups in comparison with the control using 2^−ΔΔCT^ method.

**TABLE 1:** Gene information for RT-qPCR validation.

Gene name	Length	fpkm	Read counts	Primer sequence (5′-3)
CD83	2,240	CY:36.4 LbGp:22.35 NC:52.43	CY:1,219; LbGp:3,008.5; NC:2082.3	F:CCTCTGCTCTTCACCCTG
				R:GCTATTCTGTCGCCAACT
CCL4	756	CY:3.11 LbGp:1.405 NC:8.13	CY:128.17; LbGp:199.5 NC:153.3	F:TTGTTCTCGCTCTTCTCA
				R:GTGCTGTAGTGCCTCTGG
PSPH	931	CY:2.20 LbGp:11.19 NC:1.68	CY:230.3 LbGp:77.7 NC:95.3	F:CGCAGTGTGCTTTGATGT
				R:GTCCTAGTCGTGCCGTTA
LPIN2	3,225	CY:5.09 LbGp:7.17 NC:3.29	CY:3,811.33 LbGp:2,766.83 NC:3,156.17	F:CAAGCGAAGTCATCACCA
				R:TCCACTATCAGCAGCAGC
ULK2	3,006	CY:0.03 LbGp:0.08 NC:0.038	CY:7,484.67 LbGp:3,712.3 NC:5,746.5	F:CAGCGAAACAGCACCTAT
				R:GAGTGTCTCACAACCCCA

Note: F means Forward primer, R means Reverse primer.

### 2.7 UHPLC-triple-TOF-MS

The sample is from Part 2 and used to imetabolic analysis. Ultra-high-performance liquid chromatography (UHPLC) was conducted on an ACQUITY UPLC HSS T3 column (100 mm × 2.1 mm i.d., 1.8 µm) from Waters, with the column temperature set at 40°C. The mobile phase consisted of a water-acetonitrile (95:5, v/v) solution containing. The mobile phase A consisted of 0.1% formic acid in water and 47.5% acetonitrile, mobile phase B consisted of 47.5% isopropanol and 5% water (containing 0.1% formic acid). The gradient elution programme was set as follows: 0–2.5 min, The mobile phase B concentration was maintained at 0% for the first 2.5 min, then increased to 25% for 2.5 min, before reaching 100% for 9 min. The mobile phase B concentration was then decreased to 100% for 13 min, before reaching 0% for 13.1 min, before finally reaching 0% for the remaining 16 min. The flow rate was 0.40 mL/min, and the injection volume was 10 μL. Mass spectrometry conditions: Mass spectrometry was conducted on an AB SCIEX-Triple TOF 5600 mass spectrometer with a point-spray ionisation temperature of 550°C, positive and negative voltages of 5000 V and −4000 V, respectively, and ionisation gases G1, G2, and curtain gas of 50.50 and 30 psi, respectively.

The ion pairs were scanned and detected according to the optimised declustering voltage (80 V) and collision energy (40 + 20) (range 50–1,000, cycle time was 510 ms) using a triple quadrupole (Qtrap). The values were 50 and 30 psi, respectively.

The ProgenesisQ software (Waters Corporation, United States) was employed to eliminate eigenvalues with missing values exceeding 20% within each group. These missing values were replaced with very small values and normalized by sum. Subsequently, variables with relative standard deviations exceeding 30% in the OC samples were excluded and log10 logarithmically normalized. This process was completed to gain the data required for subsequent analyses. The Ropls package was employed for principal components analysis (PCA), partial least squares discriminant analysis (PLS-DA), and orthogonal partial least squares (OPLS). Discriminant analysis (orthogonal partial least-squares discrimination analysis, OPLS-DA) was performed in conjunction with a screening of differential metabolites based on *t*-test and OPLS-DA results (P< 0.05, VIP>1). The Pathway Analysis Module (PAM) and Joint Pathway Analysis (JPA) in MetaboAnalyst 5.0 were employed to investigate the impact of LBP on the metabolic pathways in the spleen of immunosuppressed chicks.

### 2.8 Data statistics and analysis

The data from Part 1 reflect multiple (at least three) independent trials and are quantified as average values with standard error margins. Statistical analysis was performed using Prism 10.2.3 (GraphPad, Inc.), applying two-tailed t-tests at a threshold of *P < 0.05* for significance.

## 3 Results

### 3.1 Effect of LBGpH on CTX immunosuppressed chickens

The experimental design was carried out in accordance with the methodology outlined in Figure 1A. The results demonstrated that the body weight (Figure 1B), spleen index (Figure 1C), peripheral blood lymphocyte A490 nm value (Figure 1D) and the number of splenic lymphocyte germinal center (Figure 1E) were significantly affected by the LbGpH treatment.The body weight, spleen index, and peripheral blood lymphocyte A490 nm value of the chicks in the NC group, LbGpH, LbGpM, and LbGpL groups on the 7th, 14th, 21st, and 28th days were higher than that of the YC group, which suggests that the model was effectively constructed and that LBP has an opposing effect on immune suppression. The body weight, spleen index, and peripheral blood lymphocyte A490 nm values of chicks in the LbGpM group were found to be higher than those of the LbGpH and LbGpL groups at the same time point, indicating that the effect of LbGpM was more pronounced. The weight of the chicks increased over time (Figure 1B). On day 14, the LbGpM group exhibited a notably greater body weight compared to the CY group (P < 0.05). By day 21, this weight gain was not only highly significantly greater than the CY group but also significantly exceeded that of the LbGpH group. Additionally, at the 14th, 21st, and 28th days, the LbGpM group demonstrated a significantly greater weight compared to the CY group (P < 0.05). On the 21st day, the A490 nm absorbance values of peripheral blood lymphocytes for both the LbGpH and LbGpM groups were notably elevated compared to those of the NC and CY groups (P < 0.01). By day 28, the A490 nm absorbance readings of peripheral blood lymphocytes in the LbGpH and LbGpM groups were significantly greater than those observed in the LbGpL, NC, and CY groups (P < 0.01) (Figure 1D).

On the Figure 2, On the 7th day after the initial administration of the drug, lymphocytes were distributed evenly across all groups. By the 14th day, the number of germinal centres in the NC group was approximately 25–30, while the number in the CY group was approximately 20. This corresponded with a reduction in germinal centres, blurring of the marginal zone, and widening of the splenic solid. In contrast, the number of germinal centres in the LbGp groups was approximately 40–50, which was significantly higher than that of the NC and CY groups (P< 0.05). By the 21st day, the count of germinal centers in both the NC and LbGp groups was roughly 30, a figure that was markedly higher compared to the LbGp groups (P < 0.01). Similarly, on day 21, the count of germinal center in the NC and LbGp groups was around 30-40, contrasting with the CY group where it ranged from approximately 25–30. This was significantly lower than that in the NC and LbGp groups (P < 0.05). Furthermore, the germinal center were narrower and wider than those in the NC and LbGp groups. By the 28th day, the count of germinal centers across all groups ranged from 30 to 40, with no significant differences observed among the various subgroups (P > 0.05). The results indicate that the concentration of LbGpM is more appropriate, and that the test cycle can be extended to 28 days.

### 3.2 Pathways and genes of spleen immune disorders induced by LBP modulation of CTX

**FIGURE 3:**
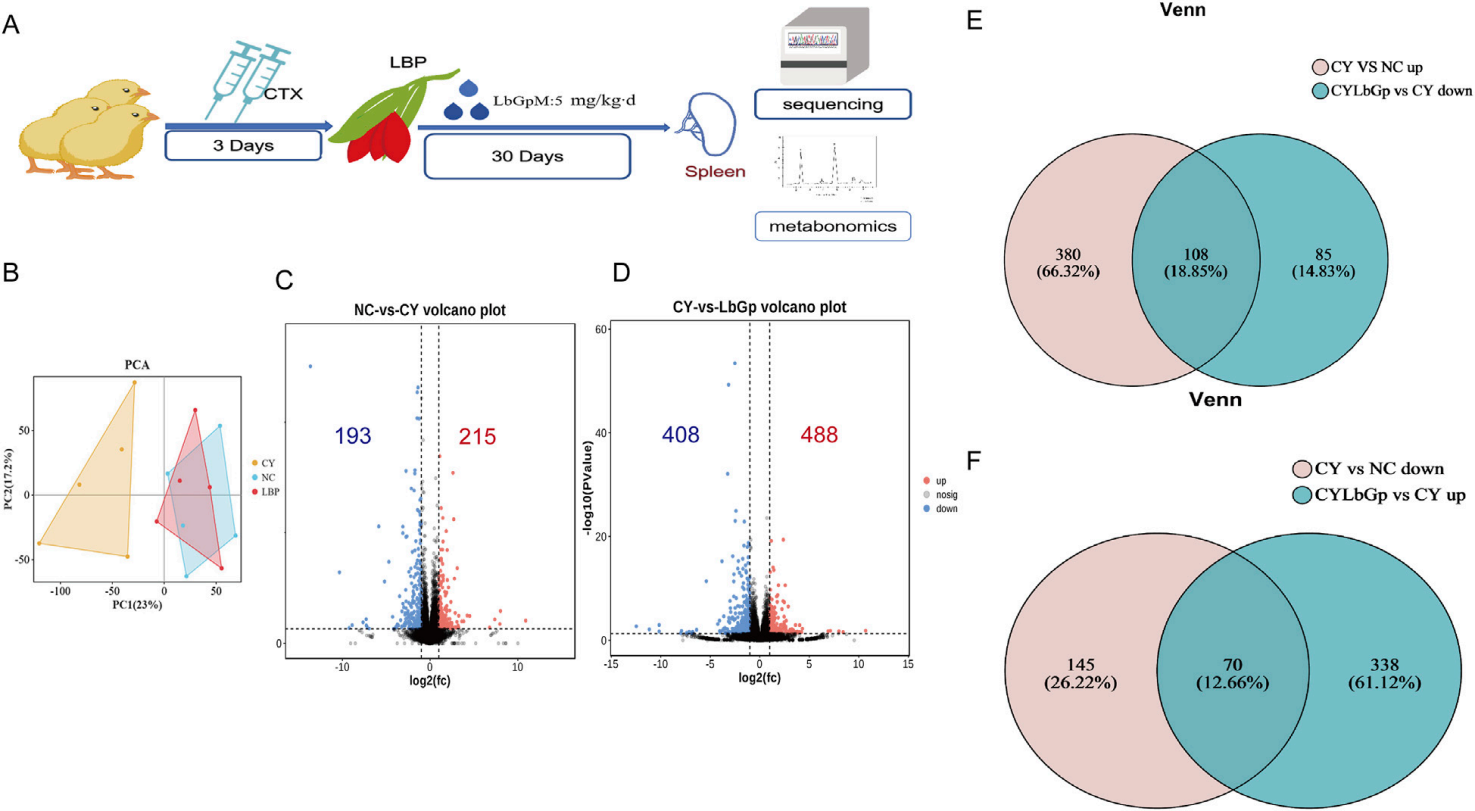
Analysis of transcriptome sequencing results. **(A)** Experimental design of Part 3. **(B)** PCA analysis results. **(C)** up-regulated and down-regulated gene volcano map in NC-CY group. **(D)** up-regulated and down-regulated gene volcano map in CY-LBGP group. **(E)** Intersection Venn map of CY vs. NC up-regulated genes and LbGp vs. down-regulated genes. **(F)** Venn diagram of intersection of CY vs. down-regulated genes and LbGp vs. CY up-regulated genes.

**FIGURE 4:**
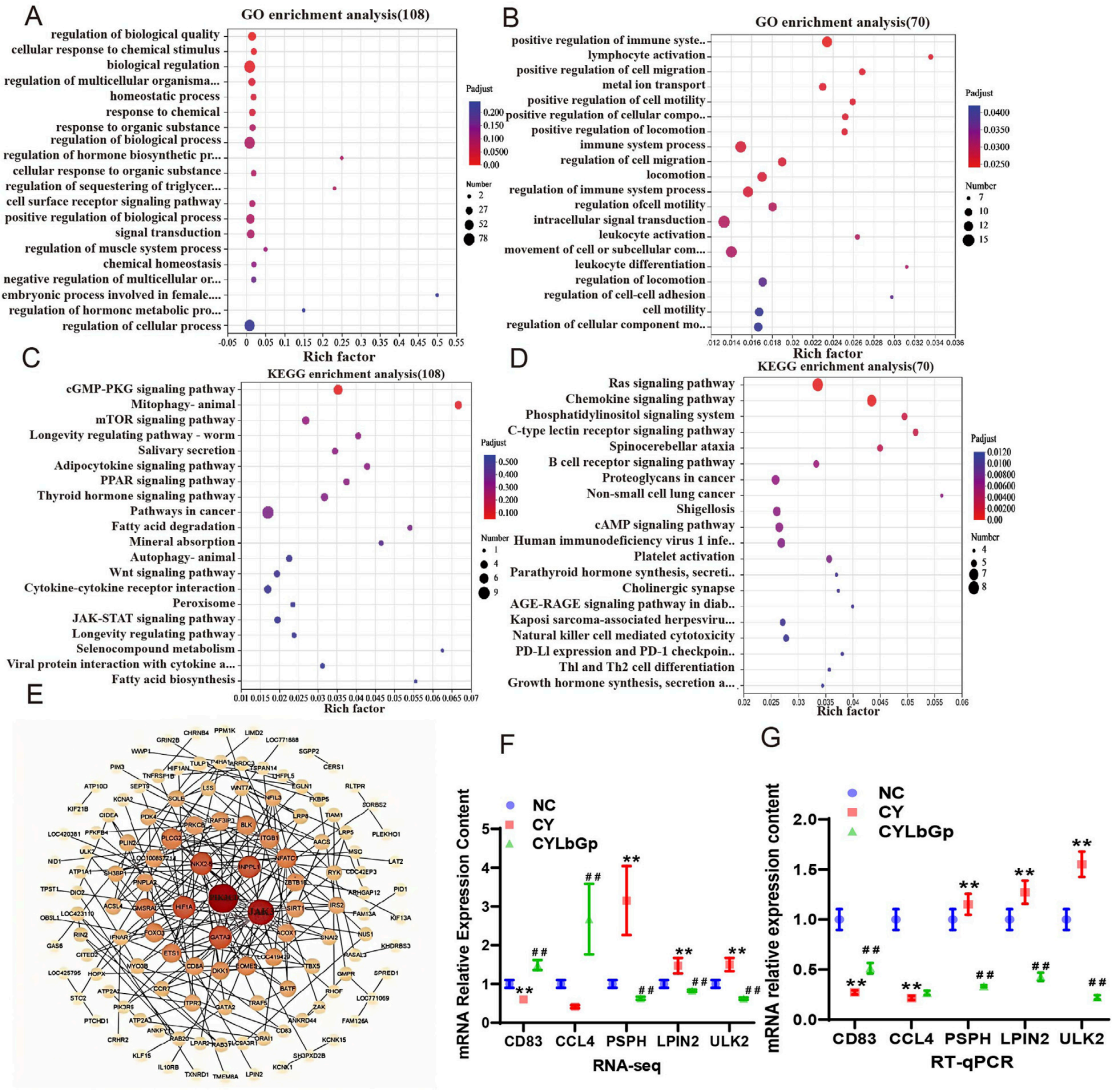
Differential gene analysis of the callback. **(A)** GO analysis of the intersection genes of CY vs. NC up-regulated genes and LbGp vs. CY down-regulated genes. **(B)** GO analysis of the intersection genes of CY vs. down-regulated genes and LbGp vs. CY upregulated genes; **(C)** CY vs. KEGG analysis of intersection genes of NC up-regulated genes and LbGp vs. CY down-regulated genes. **(D)** KEGG analysis of intersection genes of CY vs. down-regulated genes and LbGp vs. CY up-regulated genes. **(E)** PPI network map of reregulation genes. **(F)** Relative mRNA expression levels, date from RNA-seq. **(G)** Relative mRNA expression levels, date from RT-qPCR.

To further elucidate the operational mechanism of LBP, we conducted the test according to Figure 3A. To explore gene expression differences across samples and groups, PCA was performed with DESeq2 software. The results demonstrated that the gene expression profiles of the CYLbGp and NC groups exhibited substantial overlap, indicating that gene expression levels between these two groups were not significantly different. In contrast, the CY group exhibited a greater intergroup difference than the samples of the NC and CYLbGp groups, with a significant change in the gene expression levels (Figure 3B). This suggests that CTX altered the gene expression profiles of the chicken spleen, and that LBP had a significant ameliorating effect on the effects produced by the CTX stimulation. A differential expression analysis was performed on the CY group’s gene expression data, focusing on genes with a fold change ≥2 and a *p*-value <0.05. This analysis revealed the presence of 408 differentially expressed genes, of which 193 were upregulated and 215 were downregulated in comparison to the NC group. A total of 896 differential genes were identified in the CYLbGp group in comparison to the CY group. Of these, 408 were found to be upregulated, while 488 were downregulated (Figures 3C, D). In order to elucidate the principal target genes of LBP that counteract the immunosuppressive effect of CTX, the upregulated differential genes in the CY group in comparison with the NC group were selected to intersect the downregulated differential genes in the CYLbGp group in comparison with the CY group. This yielded 108 differential genes. The downregulated differential genes in the CY group compared with the NC group were intersected with the upregulated differential genes in the CYLbGp group compared with the CY group, resulting in 70 differential genes (Figure 3E, F).The 108 genes that were downregulated following the administration of the LBP intervention were significantly enriched to six GO entries that were enriched on biological processes (BP). These included regulation of biological quality, cellular response to chemical stimulus, cellular response to chemical stimulus, biological regulation, regulation of multicellular organismal process, homeostatic process, and chemical response to chemical (Figure 4). The 70 genes that were downregulated after CTX stimulation and upregulated after intervention with the administration of LBP were also significantly enriched on 23 GO BP entries (Padiust <0.05). A significant proportion of these entries were related to immune regulation, including positive regulation of the immune system process, lymphocyte activation, immune system process, immune system process, regulation of the immune system process, leukocyte activation, leukocyte differentiation, and regulation of leukocyte cell-cell adhesion. LBP represents a biological process of leukocyte activation, leukocyte differentiation, and regulation of leukocyte cell-cell adhesion (Figure 4B). The 108 differential genes that underwent downregulation and 70 upregulation following LBP intervention were subjected to KEGG pathway enrichment analysis. The results demonstrated that the 108 differential genes underwent upregulation following CTX stimulation, with 13 significantly enriched pathways identified. These pathways were predominantly associated with mitochondria, the mTOR signalling pathway, the AMPK signalling pathway and other pathways linked to cellular autophagy (Figure 4C). The 70 differential genes exhibited a marked enrichment in pathways that underwent upregulation following the administration of the LBP intervention. These pathways were predominantly associated with immune-related signalling, including the chemokine signalling pathway, the C-type lectin receptor signalling pathway, the B-cell receptor signalling pathway, platelet activation, natural killer cell-mediated cytotoxicity, and Thl and Th2 cell differentiation (Figure 4D). A total of 12 differential genes were identified as enriched in the aforementioned pathways. A protein-protein interaction (PPI) network diagram of the differential genes that were identified as being involved in the LBP intervention (Figure 4E). The results demonstrated that PIK3CD, JAK3, GATA3, HIFIA, NKX2-5 and INPPL1 were the pivotal genes, with the exception of NKX2-5, which were all enriched in the aforementioned screened differential pathways. These genes may potentially act as LBP polysaccharides to antagonise the immunosuppression of chicks.

The results of the RT-qPCR validation demonstrated that CD83, CCL4, and LBP card prognosis exhibited an upregulation trend, while PSPH, LPIN2, and ULK2 exhibited a downregulation trend. This is in alignment with the observed changes in the transcriptomics data (Figures 4F, G), indicating that the transcriptomics data are highly reliable.

### 3.3 The regulatory effect of LBP on spleen metabolism under immune suppression

**FIGURE 5:**
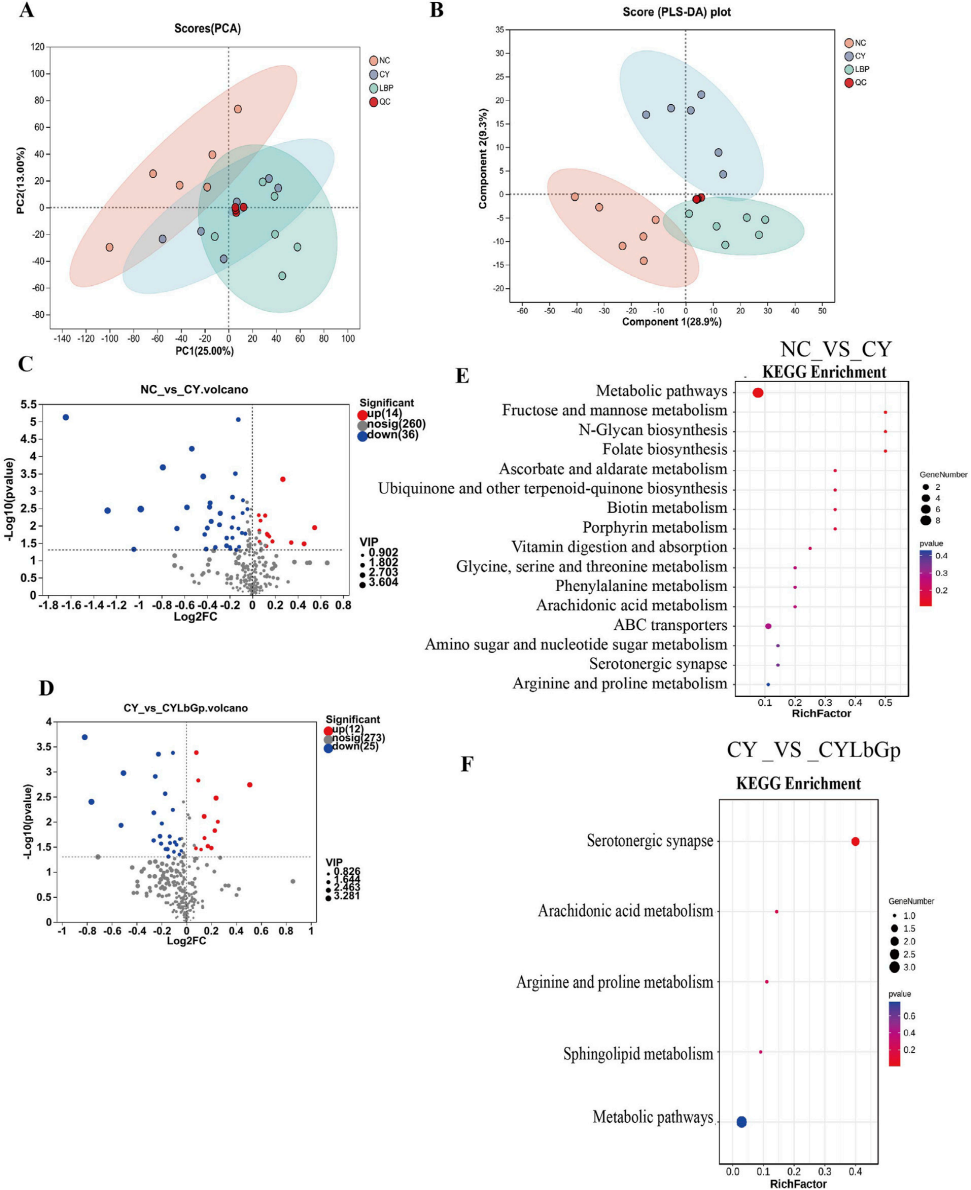
Metabolomic analysis. **(A)** PCA analysis. **(B)** PLS-DA analysis. **(C)** The volcano plot of differential genes between NC and CY. **(D)** The volcano plot of differential genes between CY and CYLbGp. **(E)** KEGG enrichment analysis of differential genes between NC_VS_CY. **(F)** KEGG enrichment analysis of differential genes between CY_VS_CYLbGp.

To delve into LBP’s impact on spleen metabolism during immune suppression, we employed UHPLC-Triple-TOF-MS technology to measure metabolites in CY, CYLbGp, and NC groups. The results revealed that the PCA plot showed changes in the spleen metabolome of the CY and CYLbGp groups compared to the NC group 30 days after drug administration, indicating that cyclophosphamide has a significant impact on the chicken spleen metabolome (Figure 5A). The metabolic profiles of the CY group and the CYLbGp group diverge at a distinct angle, indicating that there are variations in spleen metabolites between these two groups. To delve deeper into the mechanisms by which *Lycium barbarum* polysaccharide (LBP) combats immunosuppression in young chickens, a partial least squares-discriminant analysis (PLS-DA) was applied to the data from the NC, CY, and CYLbGp groups. The OPLS-DA score plot revealed a distinct separation among the three groups along the vertical axis. Notably, the samples from the NC and CY groups were located in the lower left and upper right quadrants of the plot, respectively, with the CYLbGp group’s samples positioned in between. The CY group was markedly set apart from the NC group, while the CYLbGp group’s samples intersected with both, but were closer to the NC group. This suggests that *Lycium barbarum* polysaccharide (LBP) plays a regulatory role in spleen metabolism among immunosuppressed chicks (Figure 5B).

Metabolites were selected for their differential abundance based on a VIP score above 1 and a P-value below 0.05, and these were depicted in a volcano plot. In the comparison between the NC and CY groups, a total of 310 metabolites were found to be significantly different, with 14 being upregulated and 36 downregulated in the spleen of the NC group in contrast to the CY group (refer to Figure 5C). When analyzing the CYLbGp group in relation to the CY group, 310 metabolites were noted, with 12 showing increased levels and 25 showing decreased levels in the CY group (Figure 5D). Using the KEGG enrichment method, the metabolic pathways associated with differential metabolites in each group were analyzed based on the differential metabolites’ results. The top 20 pathways, ranked by ascending P-value, are presented. The findings indicate that the differential metabolites between the CY and NC groups are predominantly enriched in pathways such as Metabolic pathways, Fructose and mannose metabolism, N-Glycan biosynthesis, Folate biosynthesis, Ascorbate and aldarate metabolism, Biotin metabolism, Porphyrin metabolism, Vitamin digestion and absorption, and ABC transporters. For the differential metabolites between the CY and CYLbGp groups, the KEGG pathways include the Serotonergic synapse, Arachidonic acid metabolism, Arginine and proline metabolism, Sphingolipid metabolism, and Metabolic pathways (Figures 5E, F).

**FIGURE 6:**
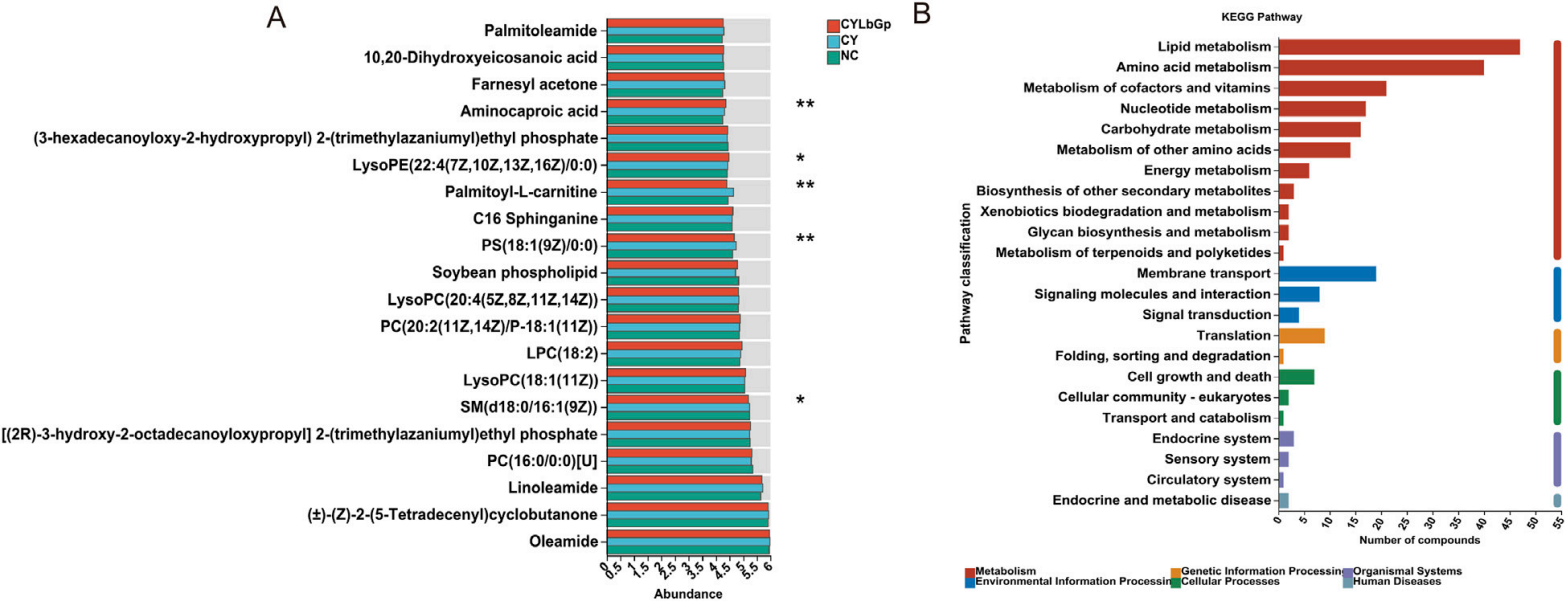
Analysis of metabolic reversed by LBP. **(A)** CY vs. NC vs. LbGp differential metabolite analysis. **(B)** KEGG enrichment analysis of 20 metabolic reversed by LBP.

To elucidate LBP’s precise influence on spleen metabolites in immunosuppressed chicks, we first compared the 114 differential metabolites between the CY and the NC, then compared them with the CYLbGp, and selected the metabolites that were reversed after LBP pretreatment, ultimately identifying 20 differential metabolites that meet the criteria (Figure 6A), mainly including Palmitoleamide, 10,20-Dihydroxyeicosanoic acid, Farnesyl acetone, Aminocaproic acid, Palmitoyl-L-carnitine, LySoPE (22:4 (7Z,10Z,13Z, 16Z) 10:0), C16 Sphinganine, PS(18:1 (9Z)/0:0), Soybean phospholipid, LysoPC(20:4 (5Z,8Z,11Z, 14Z))LysoPC(20:4 (5Z,8Z,11Z, 14Z)), PC(20:2 (11Z, 14Z)/P-18:1 (11Z)), LPC(18:2), LysoPC(18:1 (11Z)), PC(16:0/0:0)[U], SM(d18:0/16:1 (9Z)), Linoleamide (±)-(Z)-2-(5-Tetradecenyl) cyclobutanone, Oleamide. Among them, the LySOPE (22:4 (7Z,10Z,13Z, 16Z) 10:0) and Aminocaproic acidin the CYLbGp is significantly higher than CY and NC (P < 0.05), while Palmitoyl-L-carnitine, PS(18:1 (9Z)/0:0) is significantly lower than in the CY (P < 0.01), and SM(d18:0/16:1 (9Z)) is significantly lower than in the CY group (P < 0.05), indicating that LBP may exert its effects by upregulating LySOPE (22:4 (7Z,10Z,13Z, 16Z) 10:0), Aminocaproic acid, downregulating Palmitoyl-L-carnitine, PS(18:1 (9Z)/0:0), and SM(d18:0/16:1 (9Z)).

For a more comprehensive understanding of the impact of the 20 differential metabolites, MetaboAnalyst 5.0 was utilized to perform pathway enrichment analysis. This revealed several key metabolic pathways that are influenced by LBP in the spleens of chicks experiencing immunosuppression. These pathways include Lipid metabolism, Amino acid metabolism, Cofactor and vitamin metabolism, Nucleotide metabolism, Carbohydrate metabolism, Additional amino acid metabolism, Energy metabolism, and the Biosynthesis of secondary metabolites. The pathways also cover Environmental information processing, such as Membrane transport, Signaling molecule interactions, and Signal transduction. Genetic information processing involves Translation, Protein folding, sorting, and degradation. Cellular processes like Cell growth and death, and Eukaryotic cellular communities are also included. Lastly, Organismal systems such as the Endocrine system and Sensory system, as well as Human Diseases like Endocrine and metabolic diseases are implicated (as shown in Figure 6B). This indicates that LBP potentially aids in the recovery of spleen function in immunosuppressed chicks by modulating Lipid metabolism, Membrane transport, Translation, Cell growth and death, the Endocrine system, and Endocrine metabolic diseases.

### 3.4 Correlation analysis

**FIGURE 7:**
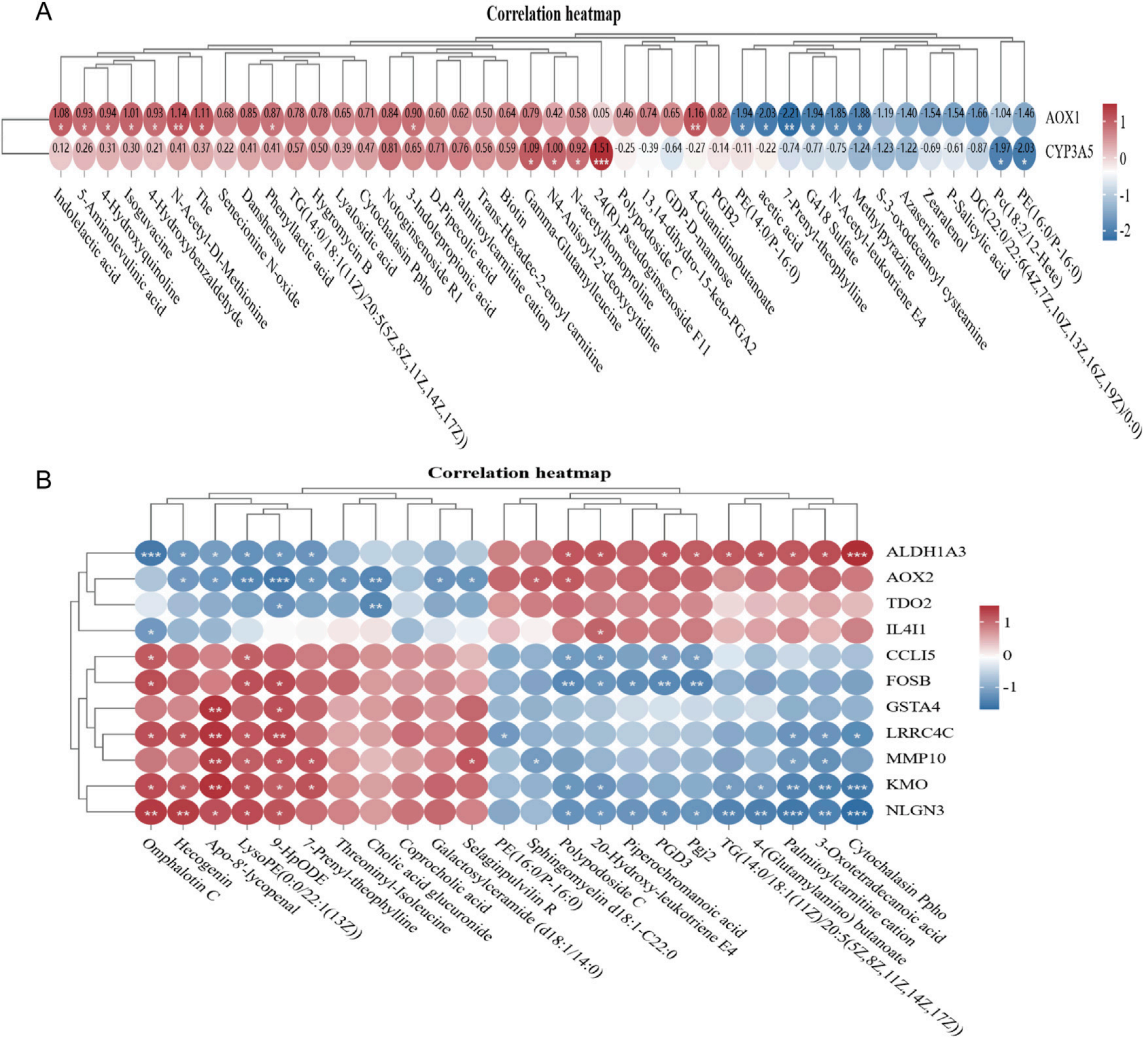
Transcriptome and metabolome association analysis **(A)**. Correlation between NC differential gene and differential metabolite in CY vs. control group **(B)**. Correlation between CYbGp vs. CY differential gene and differential metabolite.

The 178 differential genes and 20 differential metabolites were subjected to joint pathway analysis using the MetaboAnalyst 5.0 online software. Pathway impact values >0.2, a P-value <0.05, and an -log(P) value >1.3 were defined as significant metabolic pathways (Figure 7A). AOX1 and CYP3A is a key gene for CY action and regulation of metabolism (Figure 7A). The glycerophospholipid metabolic pathway is of particular significance. The key genes and key metabolites in the glycerophospholipid metabolic pathway are presented in (Figure 7B). And, LBP regulates the metabolism of the spleen through ALDHIA3, AOX 2, TDO2, IL4I1, CCLI5, FOSB, GSIA 4, LRRC4C, MMP 10, KMO, and NLGN 3.

## 4 Discussion

The causes of immunosuppression are varied, and current treatments either have some drawbacks or require further research (Houston, 2022).There are many reasons for immune suppression in poultry. Unlike treatment plans for humans, adopting safe and effective methods to treat immune suppression in poultry not only benefits the development of the poultry industry but also contributes to human food safety. *Lycium barbarum* polysaccharide (LBP), as a natural immunomodulator that can be used as both a medicine and a food (Ding et al., 2021), Its effect on immunosuppression induced by cyclophosphamide (CY) in chicks was fully verified in this study. Research results show that LbGp can effectively reverse CY induced immunosuppression and improve the body weight, spleen index and A490 nm value of peripheral blood lymphocytes of chicks, all of which indicate the recovery of immune function (Wang et al., 2024). In different doses of LBP, the effect of LbGpM group is the most significant, and the medium dose can give full play to its immunomodulatory effect. The omics technique has been widely accepted and used to study the holistic and dynamic properties of TCM in a systematic and comprehensive way (Ma et al., 2021) Moreover, it is difficult for a single statistical method to conduct a comprehensive and systematic analysis of the pathophysiological process of immunosuppression (Zhang et al., 2022).With these factors in mind, this study designed a multi-omics approach combining metabolomics and transcriptomics to reveal the anti-immunosuppressive mechanism of LBP.

Transcriptome analysis, the study revealed the effects of LbGp on the immune system at the molecular level. It mainly involves chemokine signaling pathway, C-type lectin receptor signaling pathway, B-cell receptor signaling pathway and other immune-related signaling pathways. Pathogen recognition of C-type lectin receptor (CLR) expressed by dendritic cells is important not only for antigen presentation, but also for inducing an appropriate adaptive immune response through T helper cell (TH) differentiation. The CLR acts on its own or in synergy with other receptors, such as other CLRS, toll-like receptors, and interferon receptors, to induce trigger signaling pathways that trigger specialized cytokine programs to differentiate TH cells (Geijtenbeek and Gringhuis, 2016). The spleen of mice with benzene-induced immune dysfunction also exhibited dysregulation of the B-cell receptor signaling pathway (Qiao et al., 2023), These results indicate that LBP can activate several key immune pathways and enhance immune response. In addition, the prominent positions of key genes such as PIK3CD, JAK3, GATA3, HIF1A, NKX2-5 and INPPL1 in the PPI network map further suggest that these genes may play a central role in the immune regulation of LBP. Most of these genes are involved in cell proliferation, differentiation and metabolism (Walsh et al., 2024), Phosphatidylinositol-4,5-Bisphosphate 3-Kinase Catalytic Subunit Delta (PIK3CD) is a Protein Coding geneIt phospholipises inositol lipids and participates in the immune response (Zhao et al., 2024). lnositol Povphosphate Phosphatase like 1(INPL1), Related pathways include interleukin-2 family signal transduction and D-inositol (1,45) -triphosphate metabolism pathways (Feist et al., 2016). Janus Kinase 3 (JAK3)Non-receptor tyrosine kinases involved in cell growth, development, or differentiation. It mediates innate and adaptive immunity and plays a key role in T cell development and hematopoiesis (Gupta et al., 2023; Zareifard et al., 2023). GATA Binding Protein 3 (GATA3) is an important regulator of T cell development and plays an important role in endothelial cell biology (Trujillo-Ochoa et al., 2023). Hypoxia lnducible factor 1 subunit Alpha (HF1A) affects cell metabolism, cell survival, and angiogenesis to maintain biohomeostasis (Cao et al., 2022). These results indicate that LBP further regulates phospholipid metabolism by regulating PIK3CD and INPPL1, JAK3 and JAK3 by regulating T cell development and differentiation, and HF1A by regulating angiogenesis antagonize CY induced immunosuppression. The results indicated that *Lycium barbarum* polysaccharide could regulate the immune system in a multi-pathway and multi-target way.

The metabolomic analysis revealed the profound influence of LBP on the metabolic level. There were significant changes in spleen metabolism in CY, CYLbGp and NC groups at 30 days after treatment, especially the significant difference between CYLbGp and CY groups, indicating that LBP has a regulatory effect on CY induced metabolic disorders. Through HMDB database screening and MetaboAnalyst 5.0 pathway analysis, it was found that LBP affected several key metabolic pathways, such as glycerophospholipid metabolic pathway, which is one of the important mechanisms of its regulation of immune function (Chen et al., 2024). The discovery of 20 significantly differentiated metabolites, such as Aminocaproic acid, Aminocaproic acid, a lysine analogous that effectively inhibits fibrin degradation (fibrinolysis) for the management and treatment of acute bleeding caused by elevated fibrinolysis activity (Kaseer and Sanghavi, 2024). The results showed that LBP could regulate energy metabolism, lipid metabolism and amino acid metabolism, so as to restore the normal metabolic state of immunosuppressed chicks.

Further correlation analysis indicated that the combined regulation of differential genes and differential metabolites by LBP is one of the important mechanisms for exert immunomodulatory effects. In particular, the outstanding performance of glycerophospholipid metabolism pathway in Pathway impact and statistical significance indicates that it may be a key metabolic pathway in regulating the immune function of LBP. This close association between genes and metabolites not only reveals the complexity of the action of LBP, but also provides new insights into understanding its specific mechanism of action at the molecular level. Moreover, the role of the gut-immune axis cannot be overlooked, as the large molecular structure of polysaccharides cannot be directly absorbed by the body. Previous research has shown that Proteobacteria can enhance intestinal immunity by regulating JAK3 (Mirpuri et al., 2014). The microbiota can also modulate B-cell receptor signalling pathways and natural killer cell-mediated cytotoxicity to boost immune capacity. In this experiment, metabolism is primarily enriched in lipid metabolism (Le HH et al., 2022). The increase in LySOPE (22:4 (7Z,10Z,13Z, 16Z) 10:0) and SM(d18:0/16:1 (9Z)) after LBP treatment suggests an accumulation of fats, which could also be considered in relation to the observed increase in spleen index and the faster growth state of chicks under LBP compared to the CY group, where immune function is modulated. By integrating genomics and metabolomics data, we were able to gain a more comprehensive understanding of the regulatory effects of LBP on the immune system. This close association between genes and metabolites not only reveals the complexity of the action of barbary wolfberry polysaccharides, but also provides new insights into understanding its specific mechanism of action at the molecular level. By integrating genomics and metabolomics data, we were able to gain a more comprehensive understanding of the regulatory effects of LBP on the immune system.

These results indicate that *Lycium barbarum* polysaccharide (LBP) possesses anti-immunosuppressive effects, making it highly valuable in the poultry industry. Previous research has shown that Astragalus membranaceus, Ganoderma lucidum polysaccharides (Reishi mushroom), and Panax ginseng have immune-modulating effects and have been selected for use as immune enhancers in immunoadjuvant formulations. They are considered as candidates for immune-boosting drugs and feed additives (Ma et al., 2019; Awad et al., 2019; Xue et al., 2020). However, their high cost has limited their application in animal husbandry. In comparison to the aforementioned substances, LBP is more readily available and has a lower cost, thus offering greater advantages for use in poultry management as a feed additive, an immune enhancer, or for treating immunosuppressive diseases such as Newcastle disease. This helps to improve the disease resistance of animals and reduce medication costs. In addition, globally, *Lycium barbarum* (also known as goji berries) is cultivated with an annual production of 200,000 to 300,000 tons, predominantly in China. Considering actual applications, our research has adopted a feeding method that is more closely aligned with practical production, specifically through the addition of goji berry extracts to drinking water. Our previous research found that substandard goji berries, which account for about 10% of the total output, contain 1.63 times the polysaccharides of regular berries (Ma et al., 2015). With support from the Chinese government, these substandard products are being repurposed as additives in poultry feed to reduce costs and decrease antibiotic use, thereby enhancing the safety of poultry products. This approach fosters the sustainable development of the goji berry industry and meets the market’s demand for healthier food options.

Finally, the study also has some limitations. For instance, since the focus of this experiment was solely on the anti-immunosuppressive function of *Lycium barbarum* polysaccharide (LBP), we were unable to observe the long-term effects of LBP supplementation. Data on growth performance, egg production, and resistance to actual pathogen challenges were not included. This was due to restrictive animal ethical requirements and the nature of the pathogen inoculation environment, which prevented us from assessing the long-term impacts of resistance to actual pathogen challenges. However, the anti-immunosuppressive effects and the multi-pathway immune enhancement of LBP give us greater confidence to conduct future trials on resistance to pathogen challenges. Then, Given that LBP are administered orally and have a high molecular weight requiring the involvement of gut microbiota for absorption, supplementing data on intestinal microbes will also be a part of our further work to understand their mechanism of action.With the continuous development of genomics and metabolomics technologies, higher-throughput sequencing and analysis methods can be adopted in the future to further reveal the specific regulatory mechanisms of LBP at the molecular level. In particular, techniques such as single-cell sequencing will allow a more nuanced understanding of the effects of LBP on different immune cell types.

## 5 Conclusion

This study revealed a significant antagonistic effect of LBP on chick immunosuppression. Through transcriptomic and metabolomic analysis, LBP was found to significantly upregulate immune-related genes and metabolites and optimize immune cell signaling, thereby enhancing immune function. The medium dose of LBP works best in regulating key immune processes such as mitophagy, chemokine signaling, type C lectin receptor signaling, B cell receptor signaling, platelet activation, and natural killer cells and Th 1/Th 2 cell differentiation. These findings provide important science for the use of LBP to improve immune function in poultry and reduce aquaculture losses.

## Data Availability

The datasets presented in this study can be found in online repositories. The names of the repository/repositories and accession number(s)can be found as follow: 1. The transcriptomic data were placed in the NCBI database: Accession number: PRJNA1180284 http://www.ncbi.nlm.nih.gov/bioproject/1180284; 2. The metabolome data were placed in the metabolights database: Accession number: MTBLS11538 https://www.ebi.ac.uk/metabolights/editor/study/MTBLS11538.
